# Exome localization of complex disease association signals

**DOI:** 10.1186/1471-2164-12-92

**Published:** 2011-02-01

**Authors:** Benjamin Lehne, Cathryn M Lewis, Thomas Schlitt

**Affiliations:** 1 King's College London, Department of Medical and Molecular Genetics, 8th floor Tower Wing, Guy's Hospital, London SE1 9RT, UK; 2 King's College London, MRC SGDP Centre, Institute of Psychiatry, de Crespigny Park, London SE5 8AF, UK

## Abstract

**Background:**

Genome-wide association studies (GWAS) of common diseases have had a tremendous impact on genetic research over the last five years; the field is now moving from microarray-based technology towards next-generation sequencing. To evaluate the potential of association studies for complex diseases based on exome sequencing we analysed the distribution of association signal with respect to protein-coding genes based on GWAS data for seven diseases from the Wellcome Trust Case Control Consortium.

**Results:**

We find significant concentration of association signal in exons and genes for Crohn's Disease, Type 1 Diabetes and Bipolar Disorder, but also observe enrichment from up to 40 kilobases upstream to 40 kilobases downstream of protein-coding genes for Crohn's Disease and Type 1 Diabetes; the exact extent of the distribution is disease dependent.

**Conclusions:**

Our work suggests that exome sequencing may be a feasible approach to find genetic variation associated with complex disease. Extending the exome sequencing to include flanking regions therefore promises further improvement of covering disease-relevant variants.

## Background

While development of next-generation sequencing technologies has opened exciting new opportunities to identify disease relevant mutations, whole genome sequencing of large patient cohorts is still prohibitively expensive. Therefore, more modest sequencing-based association studies are being undertaken. One way to overcome the cost limitation is the targeted sequencing of selected genomic regions such as genes or exons using enrichment methods as reviewed by Summerer [1]. Recently, several groups have successfully identified causal mutations for monogenic disorders by sequencing all exons (the "exome") in a small number of patients [2-4]. A similar approach has been proposed for complex diseases, but how important is the exome in complex disease? Cellular processes are ultimately driven by proteins, encoded by the exons, but genetic variation leading to a disorder is not necessarily located in protein coding regions. Regulatory elements that affect gene expression can play a role and, although they tend to be clustered around genes [5,6], some of them can be many kilobases (kb) upstream or downstream of the transcribed region. Further complexity is for example added by microRNAs (miRNA) with regulatory functions; some of which have been linked to disease phenotypes [7]. Whole genome sequencing may be available in the future as sequencing costs are dropping rapidly. However, for large sample numbers it is still too costly, therefore we want to identify regions that are most likely to contain disease-related variation in the human genome. To address this question we assess how disease-associated genetic variants are distributed with respect to protein-coding genes. Our analysis is based on genome-wide association study (GWAS) data for seven common diseases genotyped by the Wellcome Trust Case Control Consortium (WTCCC) (see Table 1) [8]. We first assign every genotyped SNP to its closest gene. SNPs are then binned into 10 kb windows upstream and downstream of genes. A central gene-window contains all SNPs that fall within the transcribed region of a gene and an exon-window all SNPs that fall into a coding exon. To assess the amount of association signal within every window we determine the proportion of SNPs with a p-value smaller than a threshold α.

**Table 1:** GWAS data sets for seven diseases genotyped by the WTCCC: Number of cases and SNPs after Quality Control (QC) and the Genomic Control Inflation factor λ

	Number of cases	Number of SNPs	λ
Bipolar Disorder (BD)	1,868	391,411	1.12
Coronary artery disease (CAD)	1,929	392,632	1.07
Crohn's Disease (CD)	1,752	392,990	1.12
Hypertension (HT)	1,952	392,598	1.07
Rheumatoid arthritis (RA)	1,860	392,575	1.04
Type 1 diabetes (T1D)	1,964	392,355	1.06
Type 2 diabetes (T2D)	1,924	391,860	1.08

## Results

The WTCCC study was carried out using the Affymetrix 500K GeneChip which covers ~500,000 SNPs spread out over the human genome. To explore how much of the association signal is linked to the exome, we assign every SNP to its closest gene based on genomic localisation (Table 2); all SNPs are subsequently classified into groups according to their distance from the closest gene, i.e. "within a gene", "less than 10 kb upstream", "between 10 kb and 20 kb upstream", etc. Table 3 shows the total number of SNPs represented on the Affymetrix 500k GeneChip for every window. For each SNP we perform association tests comparing the frequency of the sequence variants between diseased and healthy individuals as described in the Methods section. In the original publication by the WTCCC careful multiple testing correction procedures were employed to ensure the identification of association signals with genome-wide significance, typically with a p-value p < 5 x10^-8 ^[9]. Due to the high number of statistical tests, many SNPs are statistically significant at p < 0.05, but do not pass this genome-wide multiple testing correction. Amongst the latter might be true associations. We assume a uniform distribution of false positive signal over all SNPs, whereas true positives might be enriched in specific locations relative to the coding sequences. Here, we (arbitrarily) define a SNP where p < α with α∈{0.1, 0.01, 0.001} as "suggestive" and analyse the distribution of "suggestive" p-values with respect to exons and genes by examining the proportion of SNPs in each sequence window achieving p-values below α. To establish the significance of the association signal for each sequence window we perform 100,000 permutations of the disease status to derive a 95% confidence interval as described in the Methods section.

**Table 2:** Summary of the SNP to gene assignment

Protein-coding genes on chromosome 1-22	20,919
Protein-coding genes after SNP to gene assignment	17,058
Protein-coding genes with SNP in transcribed regions	13,783
Protein-coding genes with SNP in coding exon	2,887
SNPs on chromosome 1-22	488,665
SNPs in transcribed regions	194,831
SNPs 100 kb upstream or downstream of transcribed region	151,984
SNPs in coding exons	3,878
average gene length (whole transcript ± 1 s.d.)	53,334 ± 112,931
average gene length of genes with SNP in transcribed region (whole transcript ± 1 s.d.)	75,928 ± 132,938
average gene length (coding exons only ± 1 s.d.)	1,747 ± 1,889
average gene length of genes with SNP in exon (coding exons only ± 1 s.d.)	2,826 ± 3,339

**Table 3:** Number of SNPs per window in the observed and permuted datasets (Crohn's Disease)

CD			observed			95% confidence interval		
	**Distance**	**total**	**p < 0.1**	**p < 0.01**	**p < 0.001**	**p < 0.1**	**p < 0.01**	**p < 0.001**

	-100	2,883	277	31	2	246 - 333	16 - 44	0 - 8
	-90	3,092	286	30	11	266 - 355	18 - 46	0 - 8
	-80	3,402	351	28	5	294 - 387	20 - 50	0 - 9
	-70	3,966	396	51	7	347 - 447	25 - 56	0 - 10
	-60	4,664	450	42	11	412 - 521	30 - 65	0 - 11
Upstream	-50	5,324	569	49	6	475 - 591	36 - 73	1 - 12
	-40	6,444	645	62	9	581 - 709	45 - 86	1 - 14
	-30	7,651	823	92	22	696 - 834	55 - 100	2 - 15
	-20	10,451	1,099	131	28	963 - 1,127	79 - 132	4 - 19
	-10	14,095	1,452	164	31	1,311 - 1,508	110 - 173	6 - 25

Gene	0	152,344	15,511	1,649	284	14,698 - 15,747	1,353 - 1,687	109 - 201

Exon	0	2,876	328	40	12	253 - 323	18 - 41	0 - 7

	10	15,064	1,550	208	38	1,403 - 1,609	119 - 184	6 - 26
	20	8,954	893	110	29	818 - 973	66 - 115	3 - 18
	30	6,860	668	75	18	620 - 752	48 - 91	1 - 14
	40	5,555	588	70	11	496 - 617	37 - 76	1 - 12
Downstream	50	4,612	444	36	5	408 - 516	30 - 64	0 - 11
	60	4,088	413	38	4	358 - 461	26 - 58	0 -10
	70	3,507	339	32	1	304 - 399	21 - 51	0 - 9
	80	3,262	330	35	8	281 - 373	19 - 48	0 - 9
	90	2,882	300	30	7	246 - 332	16 - 44	0 - 8
	100	2,601	264	46	10	220 - 301	14 - 40	0 - 8

Total		274,577	27,976	3,049	559	25,443 - 28,892	2,183 - 3,283	134 - 457

The distribution of association signal varies between diseases. Figure 1 shows the enrichment of association signal for each disease at α = 0.01 (Additional File 1: Figures S1 to S7 and Additional File 2: Tables S1 to S9 for α∈{0.1, 0.01, 0.001}).

**Figure 1:**
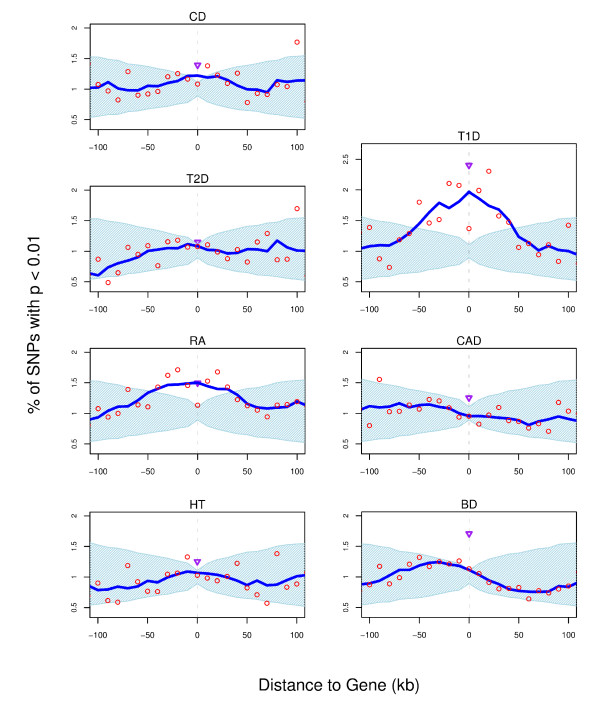
**Enrichment of association signal (p < 0.01) around the gene for seven diseases genotyped by the WTCCC**. The percentage of SNPs with p < 0.01 (red circles) is plotted for their distance to the closest gene. Values have been smoothed using a 50 kb sliding window (blue line). The light blue area represents the distribution expected by chance (95% confidence intervals) based on 100,000 permutations of the case/control status. The purple triangle represents the proportion of SNPs with p < 0.01 in coding exons.

Within genes we observe significant enrichment of association signal for Type 1 Diabetes T1D (α = {0.1, 0.01, 0.001}), Rheumatoid Arthritis RA (α = {0.01, 0.001}), Bipolar Disorder BD (α = 0.01), Crohn's Disease CD (α = 0.001) and Type 2 Diabetes T2D (α = 0.001). Coding exons typically only constitute a small fraction of the total length of a gene (~3%), but changes to the amino acid sequence are likely to alter or disrupt the function of a gene. When we therefore analyse the enrichment of association signal in exons only (purple triangles), we observe a significant enrichment of association signal for T1D (α = {0.1, 0.01, 0.001}), RA (α = {0.01, 0.001}), Hypertension HT (α = 0.1), BD (α = 0.01) and CD (α = 0.001). Furthermore, for almost all diseases and levels of α the proportion of "suggestive" SNPs in coding exons is higher than in the whole gene (except BD and T2D at α = 0.001) indicating that the majority of the association signal is to be found in the coding region rather than the introns. Outside genes, T1D, RA and CD show consistent enrichment of association around the central gene window; this enrichment becomes stronger with increasingly stringent levels of α. For the other diseases we observe sporadic enrichment.

For BD, Moskvina *et al*. compared the distribution of SNPs within and outside of genes independently of distance to the gene [10]. They reported a significant enrichment of SNPs with p < 0.01 within genes. Our analysis confirms their findings: we, too, do not find enrichment within genes for any other α for BD (Additional File 1: Figure S4), nor do we observe consistent enrichment in the vicinity of genes for BD.

**Figure 2:**
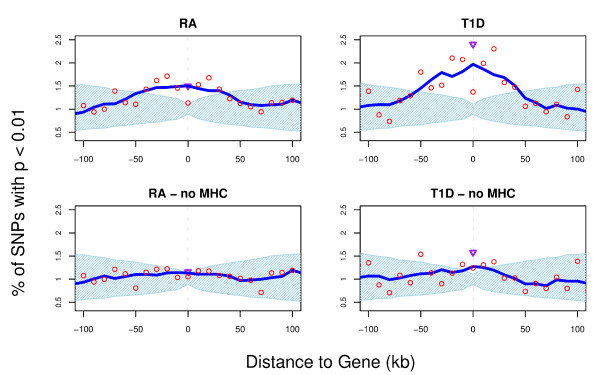
**Enrichment of association signal (p < 0.01) around the gene for RA and T1D before and after removal of the MHC region**. The percentage of SNPs with p < 0.01 (red circles) is plotted for their distance to the closest gene. Values have been smoothed using a 50 kb sliding window (blue line). The light blue area represents the distribution expected by chance (95% confidence intervals) based on 100,000 permutations of the case/control status. The MHC region was defined as chromosome 6, position 25,930,839 to 33,297,046. The purple triangle represents the proportion of SNPs with p < 0.01 in coding exons.

One factor that might be influencing our analysis is linkage disequilibrium (LD). In an ideal situation all SNPs would be inherited independently. However, SNPs located closer to each other on a chromosome are more likely to be inherited together, because the likelihood for separation due to crossing-over events is lower. When deriving confidence intervals by permuting disease labels we maintain LD structure, i.e. the confidence intervals take the LD structure into account. Yet, a true association signal could extend over large regions of the genome if it falls into a LD block. Most of the SNPs in such a region could appear to be associated with the phenotype. If such a region is rich in genes we would observe an enrichment that is inflated, because in gene-dense regions the windows close to the central gene window are more populated since every SNP is assigned to closest gene. The Major Histocompatibility Complex (MHC) region on chromosome 6 is such a gene-rich region and known to have a high level of LD. RA and T1D have their most significant signal within the MHC region. We therefore repeated our analysis but excluded the MHC region on chromosome 6 (position 25,930,839 to position 33,297,046, NCBI assembly GRCh37). For all diseases except RA and T1D the distribution changes only marginally after removal of the MHC region (Additional File 1: Figures S1 to S7); but for RA-MHC and T1D-MHC enrichment in the vicinity of genes is substantially reduced (Figure 2, Additional File 1: Figures S2 and S3). T1D-MHC continues to display a moderate but significant enrichment in and around genes and we find a significant enrichment within exons. In contrast we observe no significant enrichment for RA-MHC.

**Figure 3:**
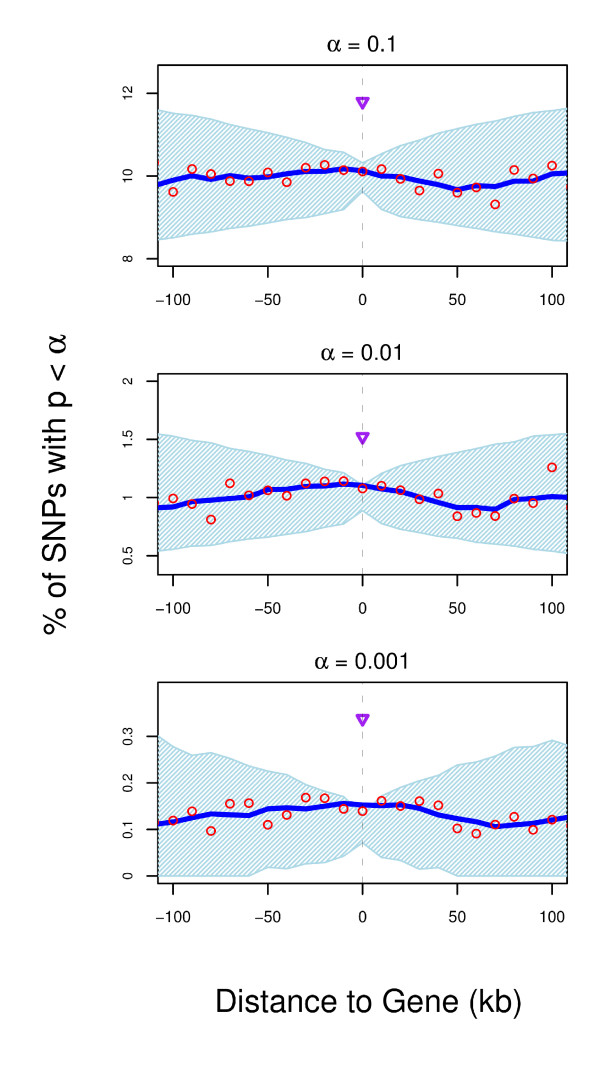
**Combined enrichment of association signal around the gene for seven diseases genotyped by the WTCCC after removal of the MHC region**. The percentage of SNPs with p < α (red circles) is plotted for their distance to the closest gene. Values have been smoothed using a 50 kb sliding window (blue line). The light blue area represents the distribution expected by chance (95% confidence intervals) based on 100,000 permutations of the case/control status. The MHC region was defined as chromosome 6, position 25,930,839 to 33,297,046. The purple triangle represents the proportion of SNPs with p < α in coding exons.

To test if we can increase the power of our analysis we combine the data for all seven diseases (Figure 3, Additional File 1: Figures S8 and S9). When the MHC region is excluded from the analysis (Figure 3 and Additional File 1: Figure S9) the combined distribution of all seven diseases shows moderate enrichment. The enrichment is still significant for α = 0.001 and we observe a bell shaped distribution around the gene for all three thresholds α (Additional File 1: Figure S9), suggesting a deviation from uniform distribution. For all thresholds α the proportion of SNPs with p < α is higher in exons than in the whole gene (purple triangle in Figure 3).

In this work we analyse the distribution of SNPs with "suggestive" p-values in respect to genes. To assess whether the observed enrichment is driven by a few genes only, we count the number of genes with SNPs that have "suggestive" p-values (p < α) (Table 4). We considered SNPs that are located within less than 100 kb of a gene and SNPs that are located within an exon separately (Table 4). Gene counts for the observed data are compared to gene counts from 100,000 permutations of the disease status. We observe significantly more genes with "suggestive" SNPs for the diseases and levels of α where we observe an increase of association signal. This suggests that the observed enrichment is not driven by a few genes only. For T2D (α = 0.001) and BD (α = 0.01) the number of genes with "suggestive" SNPs does not reach statistical significance, but is notably above the centre of the 95% confidence interval. Diseases that show an enrichment of association signal around the gene (CD, T1D, RA) also have substantially larger number of genes with "suggestive" SNPs than the diseases that do not show enrichment (T2D, CAD, HT, BD) (Table 4).

**Table 4:** Total number of genes with SNPs (after QC) and number of genes with SNPs at p < α

			observed			95% confidence interval		
		**Total**	**p < 0.1**	**p < 0.01**	**p < 0.001**	**p < 0.1**	**p < 0.01**	**p < 0.001**

	CD	16,381	6,941	1,306	222	6,712-7007	1192-1367	131-188
	T1D	16,387	7,108	1,547	389	6,709-7004	1191-1366	131-188
	T1D-MHC	16,206	6,945	1,400	254	6658-6952	1184-1358	130-187
Gene	T2D	16,375	7,002	1,289	172	6702-6999	1189-1364	130-188
± 100 kb	RA	16,381	7,064	1,493	281	6708-7003	1190-1366	131-188
	RA-MHC	16,203	6,933	1,400	203	6658-6952	1184-1359	130-187
	HT	16,382	7,057	1,328	191	6707-7003	1191-1366	130-188
	CAD	16,377	6,993	1,267	173	6704-7002	1191-1365	130-188
	BD	16,377	7,019	1,352	187	6699-6995	1188-1363	130-188

	CD	2,264	291	33	11	234-296	17-38	0-6
	T1D	2,263	316	61	24	234-296	17-38	0-6
	T1D-MHC	2,236	295	41	8	232-293	17-38	0-6
	T2D	2,259	263	30	1	234-294	17-38	0-6
Exon	RA	2,265	293	38	12	235-296	17-38	0-6
	RA-MHC	2,238	278	28	4	232-293	17-38	0-6
	HT	2,265	300	31	3	234-295	17-38	0-6
	CAD	2,260	296	34	5	234-294	17-38	0-6
	BD	2,258	281	46	2	233-294	17-38	0-6

Numbers are derived for all seven diseases and for T1D and RA after removal of the MHC region (T1D-MHC and RA-MHC). We consider SNPs that are located within less then 100 kb of a gene and SNPs that are only located within an exon. We derived 95% confidence intervals for the number of genes with p < α based on 100,000 permutations of the disease status.

## Discussion

In this work we consider SNPs that show a "suggestive" association (p-value < α with α∈{0.1, 0.01, 0.001}) with a disease. Using the genome annotation (NCBI Gene Build downloaded in November 2009, NCBI assembly GRCh37) we find these "suggestive" SNPs to be enriched in genes and their vicinity. We observe a significant enrichment of association signal in protein-coding exons (T1D, CD, HT, BD), in genes (T1D, CD, BD, T2D) and in regions up- and downstream of genes (T1D, CD). The distribution of association signal varies between the different diseases, possibly due to different genetic architectures of the analysed diseases. Yet, for all seven diseases we found a consistently stronger association signal in coding than in non-coding regions.

A major issue in our analysis and various related studies [11-20] is the presence of linkage disequilibrium (LD) which makes it very difficult to allocate association signal correctly. The causal variant might be located several kilobases from a variant in LD, which substantially complicates the identification of the causal gene. In our analysis LD might in particular inflate the enrichment for the immune related diseases RA and T1D, for which most of the association signal is located in the MHC region. We address this problem by taking into account LD structure when deriving confidence intervals and by removing the MHC region. While removing the MHC region did not influence the enrichment results for most of the diseases examined here we found profound effects on the results for T1D and RA. The WTCCC study has shown a substantial fraction of the variation associated with RA and T1D are located within the MHC region; thus ignoring this region in the enrichment analysis is likely to result in an underestimate of the true enrichment signal, while including the MHC region probably leads to an inflated signal.

Because of the way we assign SNPs to genes, the total number of SNPs per window is substantially higher within genes and their vicinity (Table 3). This is reflected by the 95% confidence interval which becomes wider with increasing distance from the central gene window. As a result the statistical power to detect a significant enrichment of association signal is higher closer to the central gene window. However, if the observed enrichment was only due to an increase in statistical power around the gene window we would expect a similar percentage of SNPs with p < α for every window, which would become significant around the gene due to the smaller confidence interval. In contrast, for all diseases that show enrichment we observe an increase of association signal around the gene window rather than just a decrease of the 95% confidence interval.

Our approach takes into account distance between variants and genes, which ultimately allows us to detect enrichment; however, effects of genomic features that have a variable distance with respect to genes (e.g. enhancers) will not be detected by our approach. In fact the WTCCC could not associate a number of replicated GWAS hits with any gene because they fall into so-called gene deserts, with the closest gene being over 100 kb away [8]. Detection and interpretation of these variants is likely to improve as the annotation of sequence elements such as miRNAs, enhancers and other regulatory features is increasingly available and some are already included in the exome enrichment kits offered by commercial suppliers. As long as annotation of non-coding elements is still sparse, sequence conservation might be a reasonable proxy for an analysis similar to the one presented here.

Our analysis of genetic association is limited by the SNPs represented on the Affymetrix 500K GeneChip, which provides exonic SNPs for ~14.5 % of all genes. Other genotyping arrays might capture association signals that are not detectable using this platform and as a consequence the distribution of association signal might differ.

In addition to common variants, sequencing allows for the detection of rare variants. Rare variants that show association with a disease might be distributed differently from the common variants analysed here. We repeated our analysis for rare SNPs (minor allele frequency of less than 0.05), but did not observe a bias towards genes or exons. This is mainly due to the lack of statistical power, because only 10% of the SNPs that pass Quality Control are rare. Little is known about the role of rare variants in genetic traits although candidate gene studies for common genetic traits have found rare alleles with strong effect sizes in coding regions [21,22] and the majority of known causal variants in mendelian disorders are non-synonymous mutations or mutations in splice sites [2].

## Conclusions

We found a consistently stronger association signal in coding than in non-coding regions for all seven diseases analysed by the WTCCC. We also observed an enrichment of association signal in the vicinity of genes which varies between diseases. We therefore recommend that sequencing efforts focus on the exome and, depending on the disease, to extend the targeted sequence to include other annotated elements, as well as regions up- and downstream of genes. The latter can be very costly. Whereas the entire exome only accounts for 2.3% of the genome, including 40 kb flanking windows would mean sequencing approximately 34% of the genome. Including 10 kb flanking window reduces the amount of sequence to 14%; focusing on annotated regulatory elements reduces it further. Until whole genome sequencing for large cohorts becomes affordable sequencing "extended" exomes seems to provide a sensible way to reduce costs while maximizing the chances of detecting disease-associated variants.

## Methods

### GWAS data quality control and association testing

GWAS of seven diseases have been reported by the WTCCC [8]. Approximately 2,000 cases and 3,000 shared controls were genotyped for every disease on the Affymetrix GeneChip 500K Mapping Array Set (Table 1). Our analysis includes moderate associations which are more susceptible to study biases. Moreover, we wanted to make our results comparable to a related study by Moskvina *et al. *[10]. We therefore re-analyzed the WTCCC I data performing very conservative Quality Control using PLINK v1.06 [23]. In addition to SNPs and individuals in the exclusion lists provided with the genotyping data, we applied more stringent quality control criteria. Based on the pooled case/control dataset we excluded SNPs with Hardy-Weinberg equilibrium p < 0.001, a minor allele frequency of less than 0.01 or call-rates of less than 0.97. Association testing was performed using an Armitage trend test (1df). We manually checked the most strongly associated SNPs for every disease to ensure consistency with the original WTCCC I results. To take into account inflated test statistics caused by population stratification we corrected χ^2 ^values using the genomic control metric λ_median _as described by Devlin and Roeder [24]. The estimated λ_median _(for simplicity denominated as λ) range from 1.04 to 1.12 (Table 1) and are in good agreement with the original values reported by the WTCCC. For every disease, 100,000 permutations of the disease status were performed using the PLINK max(T) permutation method and association p-values were calculated.

### Gene to SNP assignment

A tab-delimited text-file (seq_gene.md) containing genomic coordinates for all genes was downloaded from the NCBI ftp-server in November 2009 [25]. Only entries for the human reference sequence (NCBI assembly GRCh37) and protein-coding genes were retained. Genes mapping to sex-chromosomes, the mitochondrial chromosome, unassembled contigs or alternative haplotypes were discarded. SNPs on the GeneChip 500K Mapping Array Set were assigned to the remaining genes. Because this genotyping platform is based on the previous assembly of the human genome (NCBI 36) all SNP positions were converted to the latest assembly using the "Lift-Over" tool on the GALAXY website [26]. SNPs were assigned to a gene if they are located within the primary transcript of that gene. None of the SNPs on the Affymetrix 500K GeneChip fell into a region where the primary transcripts of two genes overlapped. All other SNPs were assigned to their closest gene and were then binned into 10 kb windows upstream and downstream of the gene. Positions for coding exons were obtained using GALAXY [26] and SNPs within coding exons were labelled as such. In total we assigned SNPs to approximately 17,000 genes. Table 2 summarises the SNP to gene assignment. We performed our analysis with and without the MHC region. Removal of the MHC region (chromosome 6, position 25,930,839 to position 33,297,046, NCBI assembly GRCh37) excluded 1,473 SNPs and 185 genes.

### Enrichment Plots

We determined the extent of association signal in every 10 kb window upstream and downstream of a gene and within genes. For each window and for coding exons we calculated the ratio of the number of SNPs with an association p-value below threshold α and the total number of polymorphic SNPs within that window. The same procedure was applied to the results of each of the 100,000 permuted data sets. Thus, we derived a 95% confidence interval for the proportion of SNPs significant at a p-value α when no association is present. To highlight the overall trend, values were smoothed for the observed data by averaging values over a 50 kb sliding window. Where results are shown for the combined datasets, the numbers of SNPs per window (observed and permuted) are averaged over all seven diseases.

## Abbreviations

BD: Bipolar Disease; CAD: Coronary Artery Disease; CD: Crohn's Disease; GWAS: Genome-wide association study; HT: Hypertension; LD: Linkage Disequilibrium; MHC: Major Histocompatibility Complex; miRNA: micro RNA; QC: Quality Control; RA: Rheumatoid Arthritis; RA-MHC: RA dataset without the MHC region; SNP: Single Nucleotide Polymorphism; T1D: Type 1 Diabetes; T1D-MHC: T1D dataset without the MHC region; T2D: Type 2 Diabetes; WTCCC: Wellcome Trust Case Control Consortium

## Authors' contributions

BL, CML and TS designed the project, BL performed the computational analysis, CML and TS supervised the research; the authors contributed equally to writing the manuscript. All authors read and approved the final manuscript.

## Supplementary Material

Additional File 1 **Enrichment Plots for all Diseases and Thresholds**. This file presents the enrichment plots for all seven diseases and thresholds α with and without the MHC region (Figure S1 to S7). Figure S8 and S9 show the combined enrichment of all seven diseases for all thresholds α with and without the MHC region. Click here for file

Additional File 2 **Enrichment Tables for all Diseases and Thresholds**. This file presents the enrichment tables for all seven diseases and thresholds α. Enrichment tables after removal of the MHC region are provided for RA, T1D and the combined enrichment of all seven diseases. Click here for file
