# Supplementary material for: Exome localization of complex disease association signals
Source: BMC Genomics. 2011 Feb 1;12:92. doi: 10.1186/1471-2164-12-92 (PMC3045337; doi:10.1186/1471-2164-12-92)
Supplement: Additional File 1 — Enrichment Plots for all Diseases and Thresholds. This file presents the enrichment plots for all seven diseases and thresholds α with and without the MHC region (Figure S1 to S7). Figure S8 and S9 show the combined enrichment of all seven diseases for all thresholds α with and without the MHC region. [file 1471-2164-12-92-S1.PDF]

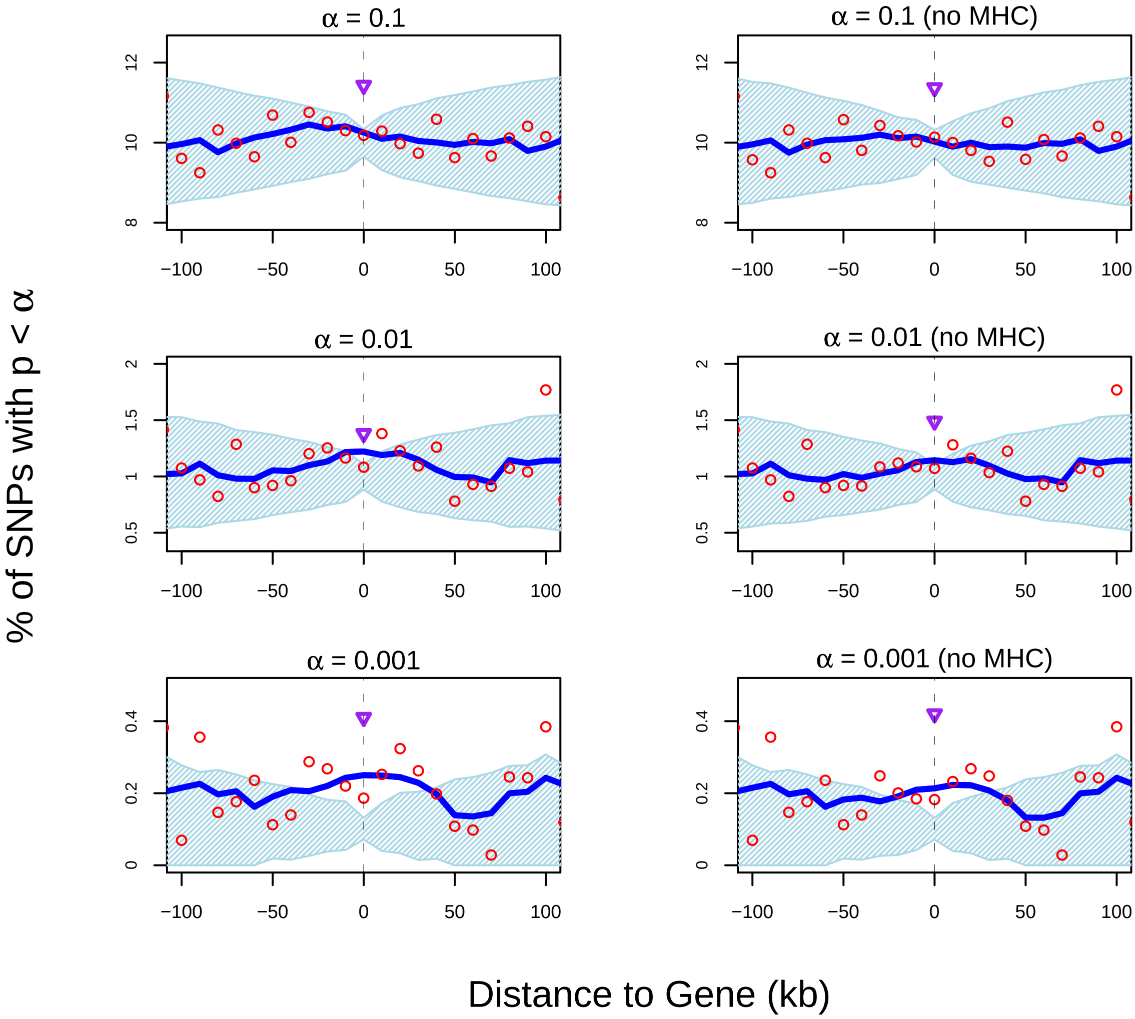

Figure S 1 : Enrichment of association signal around the gene for CD .
The percentage of SNPs with $p < \alpha$ (red circles) is plotted for their distance to the closest gene and values have been smoothed using a 50 kb sliding window (blue line). The light blue area represents the distribution expected by chance (95% confidence intervals) based on 100,000 permutations of the disease status. The purple triangle represents the proportion of SNPs with $p < \alpha$ in coding exons. For the plots on the right we excluded SNPs and genes in the MHC region.

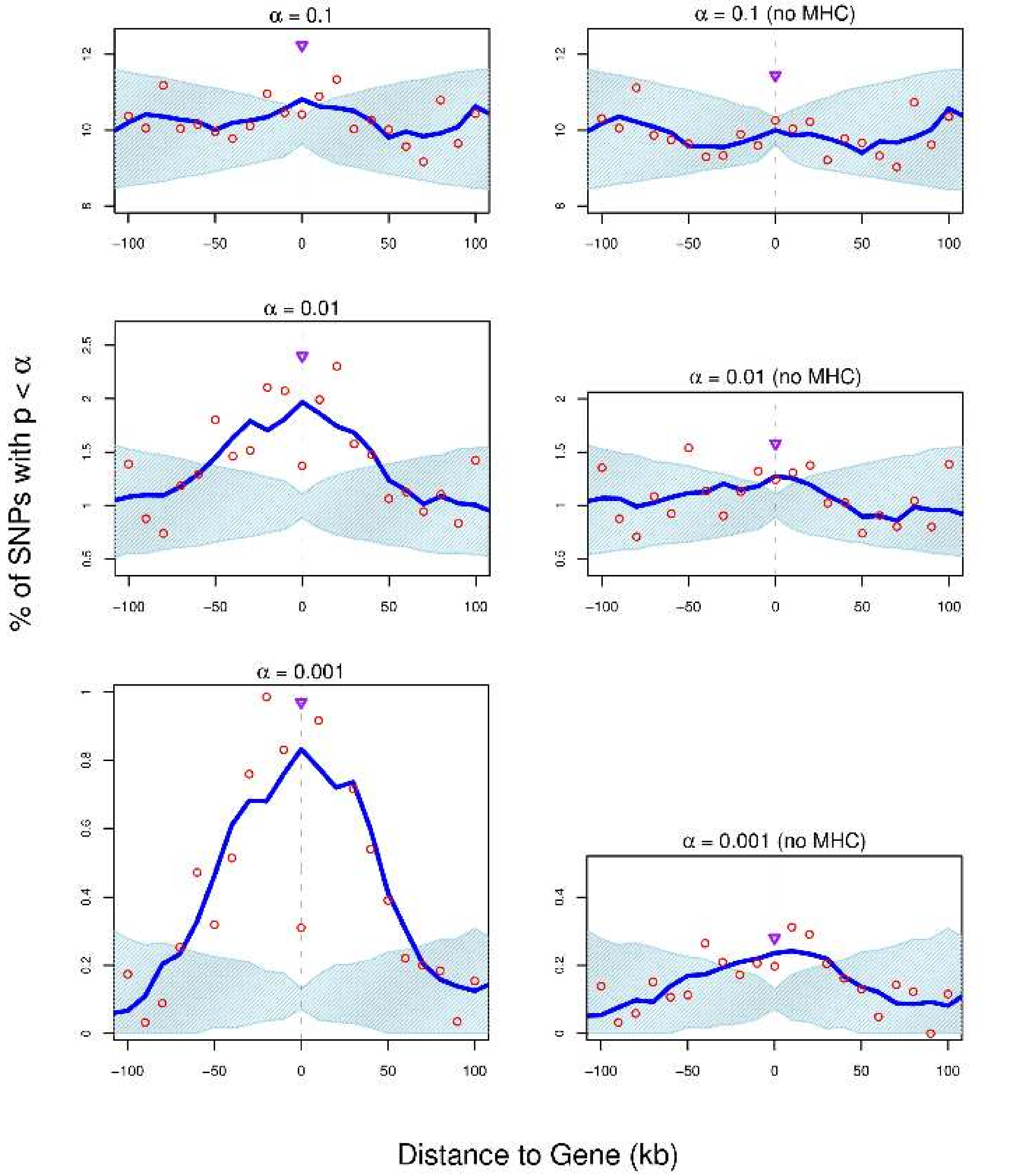

Figure S 2 : Enrichment of association signal around the gene for T1D .
The percentage of SNPs with $p < \alpha$ (red circles) is plotted for their distance to the closest gene and values have been smoothed using a 50 kb sliding window (blue line). The light blue area represents the distribution expected by chance (95% confidence intervals) based on 100,000 permutations of the disease status. The purple triangle represents the proportion of SNPs with $p < \alpha$ in coding exons. For the plots on the right we excluded SNPs and genes in the MHC region.

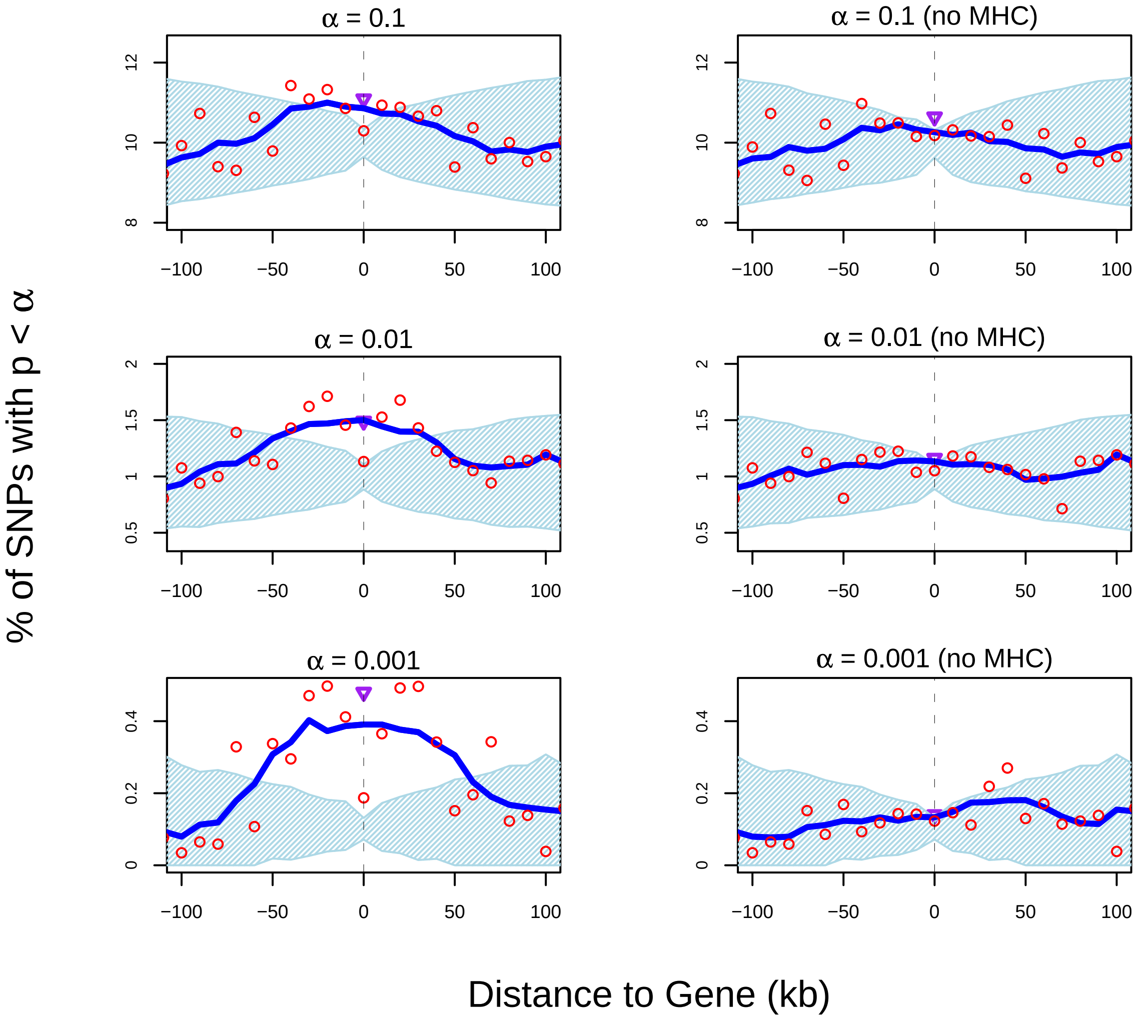

Figure S 3 : Enrichment of association signal around the gene for RA .
The percentage of SNPs with $p < \alpha$ (red circles) is plotted for their distance to the closest gene and values have been smoothed using a 50 kb sliding window (blue line). The light blue area represents the distribution expected by chance (95% confidence intervals) based on 100,000 permutations of the disease status. The purple triangle represents the proportion of SNPs with $p < \alpha$ in coding exons. For the plots on the right we excluded SNPs and genes in the MHC region.

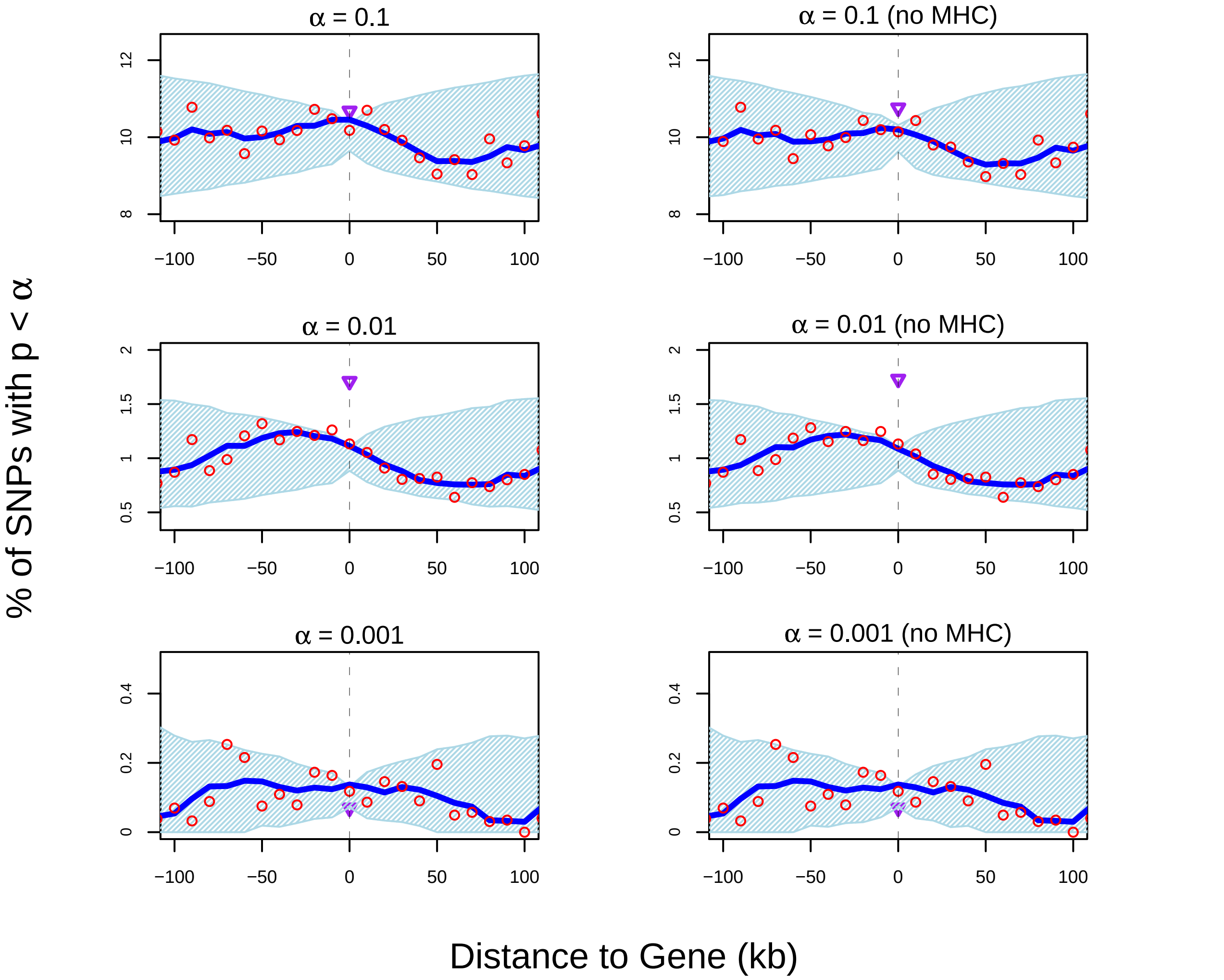

Figure S 4 : Enrichment of association signal around the gene for BD .
The percentage of SNPs with $p < \alpha$ (red circles) is plotted for their distance to the closest gene and values have been smoothed using a 50 kb sliding window (blue line). The light blue area represents the distribution expected by chance (95% confidence intervals) based on 100,000 permutations of the disease status. The purple triangle represents the proportion of SNPs with $p < \alpha$ in coding exons. For the plots on the right we excluded SNPs and genes in the MHC region.

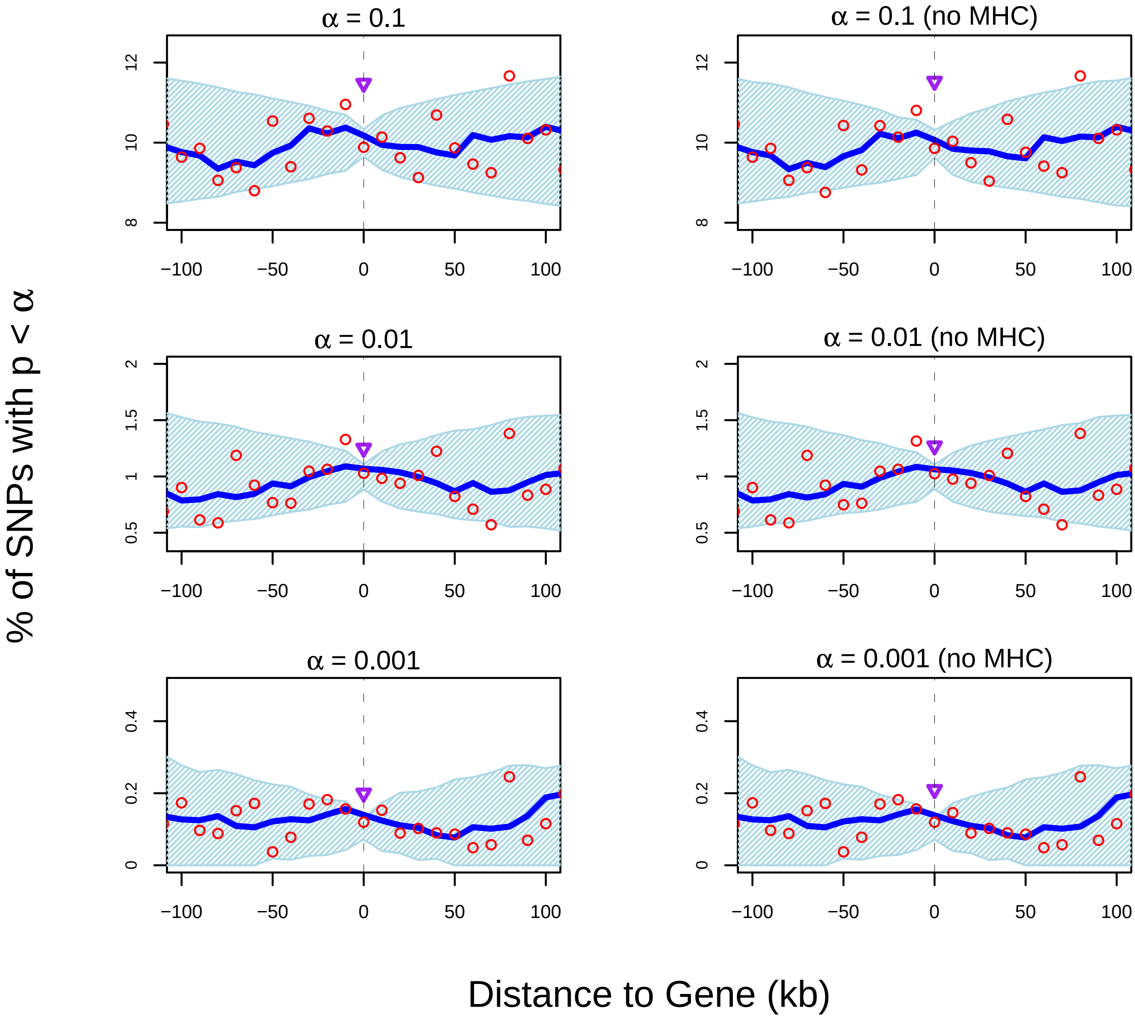

Figure S 5 : Enrichment of association signal around the gene for HT .
The percentage of SNPs with $p < \alpha$ (red circles) is plotted for their distance to the closest gene and values have been smoothed using a 50 kb sliding window (blue line). The light blue area represents the distribution expected by chance (95% confidence intervals) based on 100,000 permutations of the disease status. The purple triangle represents the proportion of SNPs with $p < \alpha$ in coding exons. For the plots on the right we excluded SNPs and genes in the MHC region.

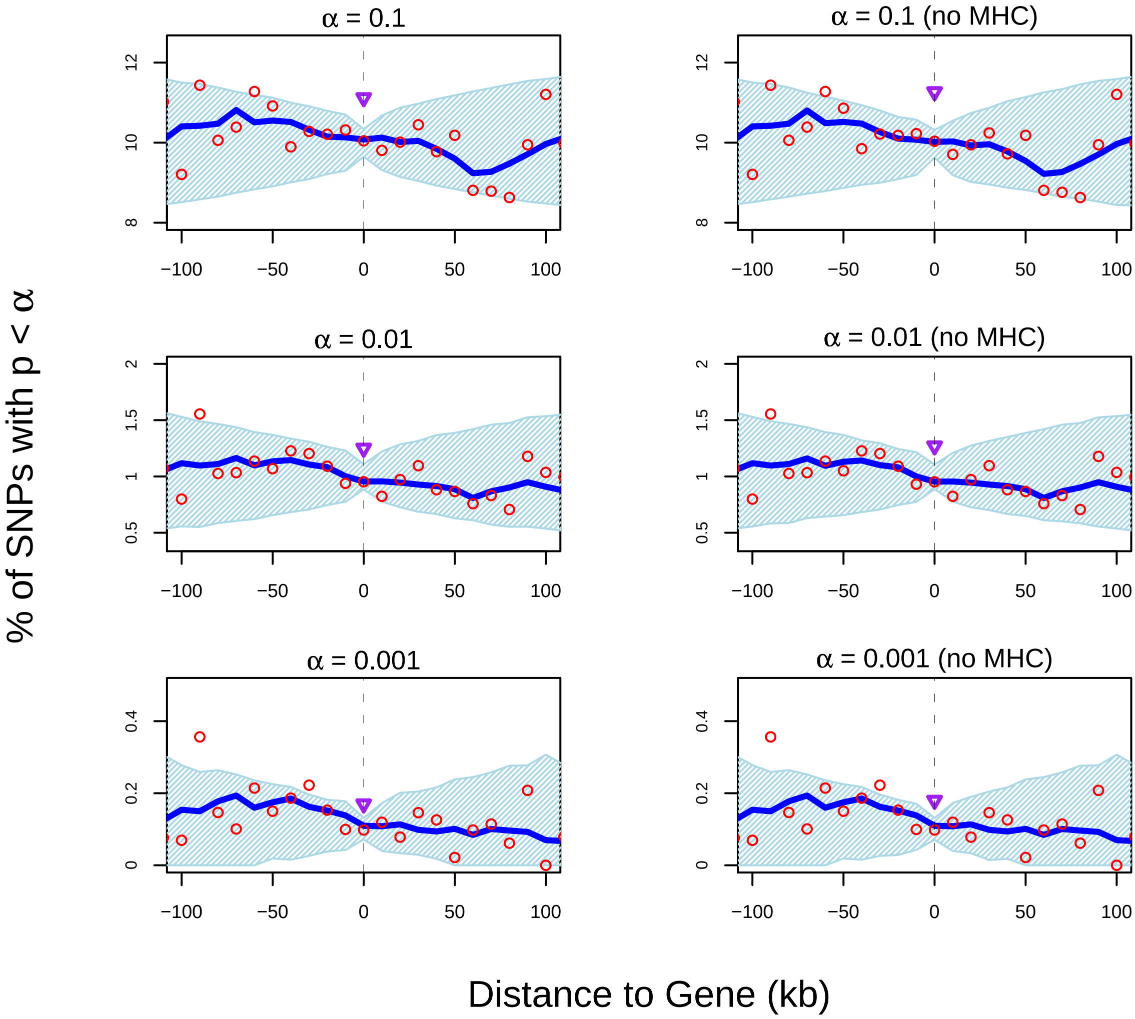

Figure S 6 : Enrichment of association signal around the gene for CAD .
The percentage of SNPs with $p < \alpha$ (red circles) is plotted for their distance to the closest gene and values have been smoothed using a 50 kb sliding window (blue line). The light blue area represents the distribution expected by chance (95% confidence intervals) based on 100,000 permutations of the disease status. The purple triangle represents the proportion of SNPs with $p < \alpha$ in coding exons. For the plots on the right we excluded SNPs and genes in the MHC region.

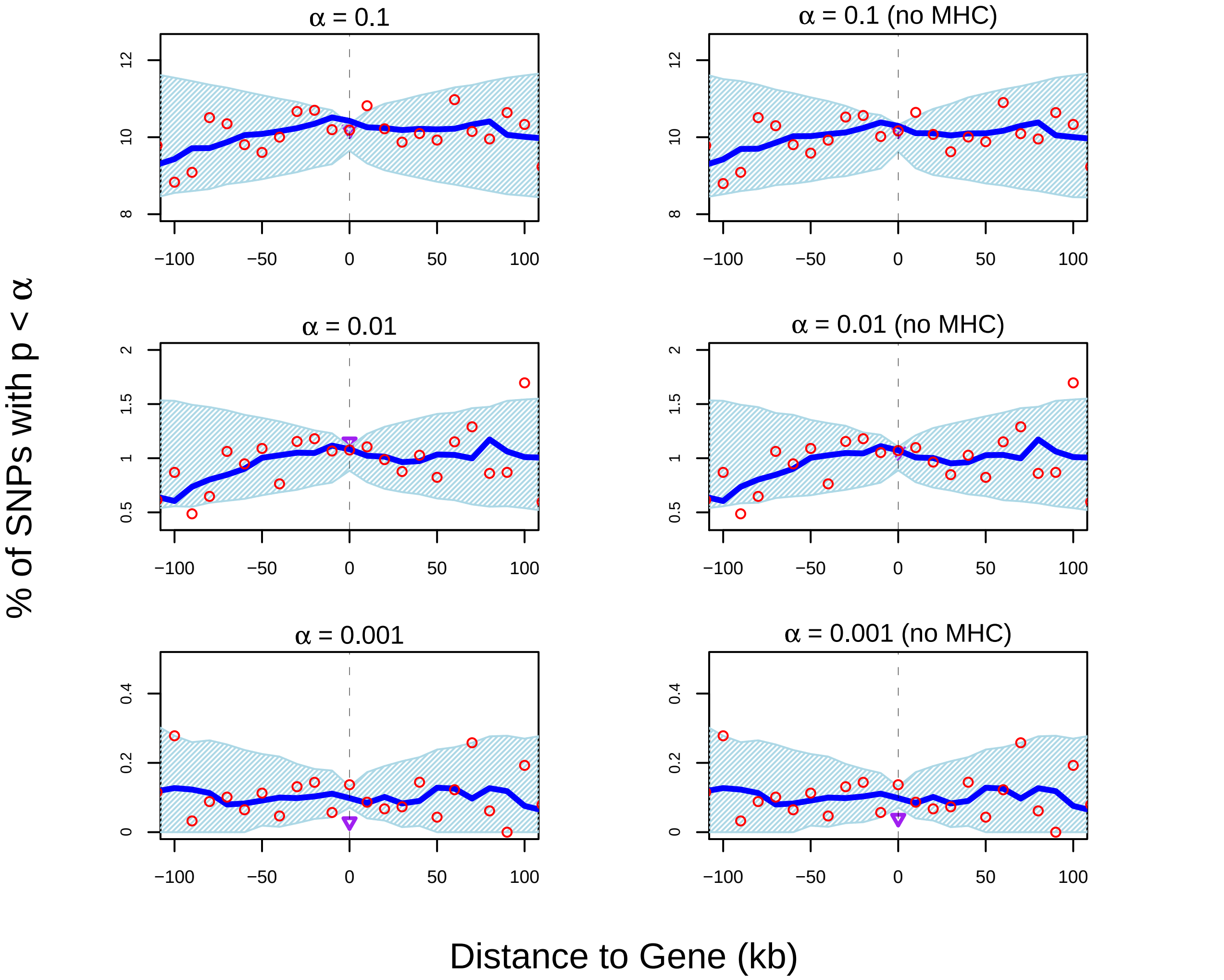

Figure S 7 : Enrichment of association signal around the gene for T2D .
The percentage of SNPs with $p < \alpha$ (red circles) is plotted for their distance to the closest gene and values have been smoothed using a 50 kb sliding window (blue line). The light blue area represents the distribution expected by chance (95% confidence intervals) based on 100,000 permutations of the disease status. The purple triangle represents the proportion of SNPs with $p < \alpha$ in coding exons. For the plots on the right we excluded SNPs and genes in the MHC region.

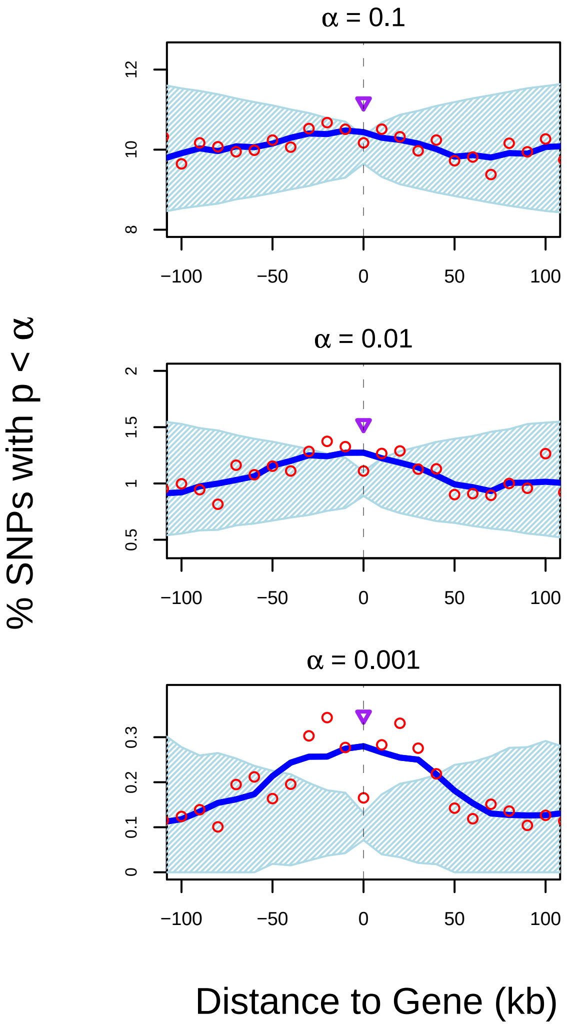

Figure S 8 : Combined Enrichment of association signal around the gene for seven diseases. The percentage of SNPs with $p < \alpha$ (red circles) is plotted for their distance to the closest gene and values have been smoothed using a 50 kb sliding window (blue line). The light blue area represents the distribution expected by chance (95% confidence intervals) based on 100,000 permutations of the disease status. The purple triangle represents the proportion of SNPs with $p < \alpha$ in coding exons.

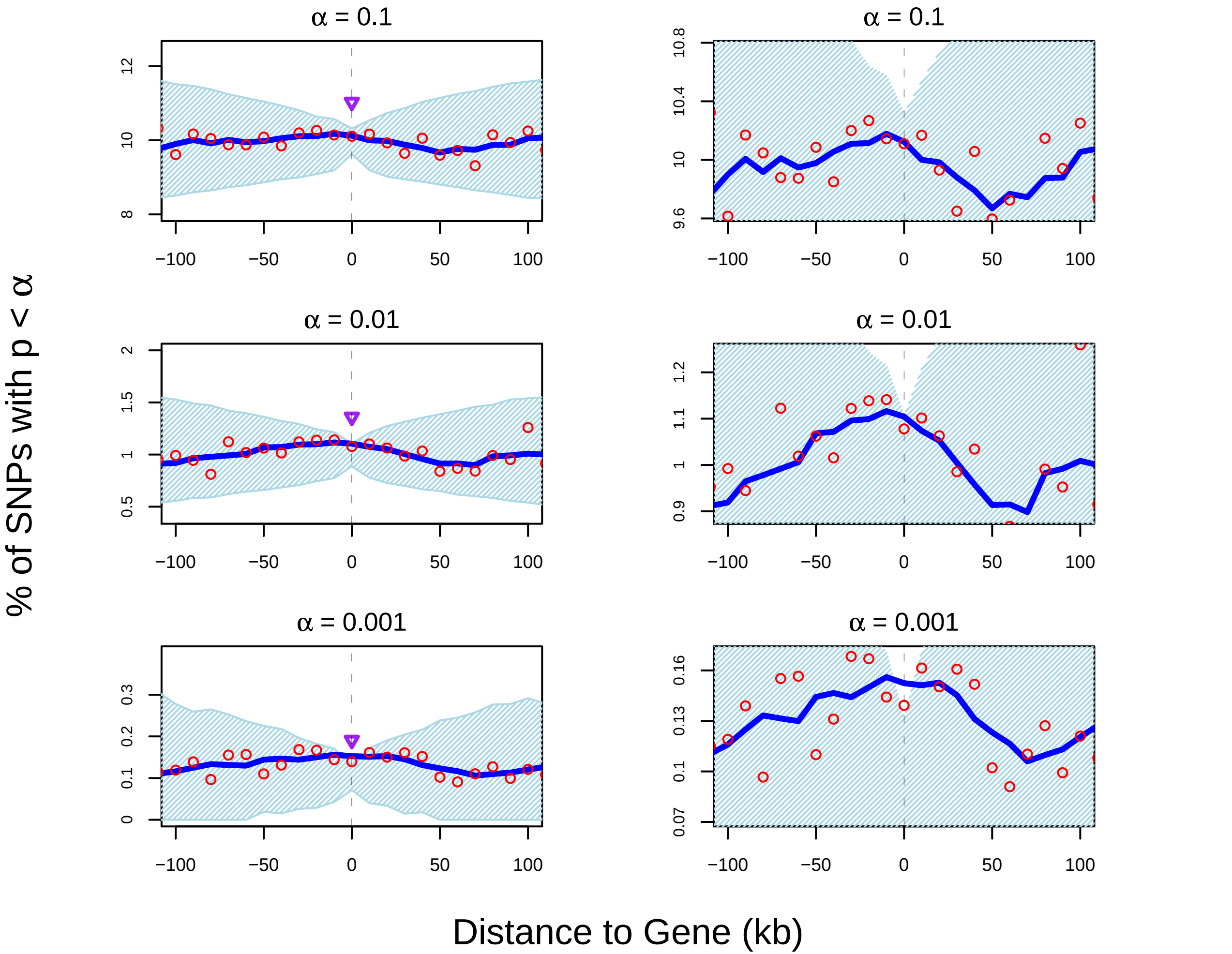

Figure S 9 : Combined enrichment of association signal around the gene for seven diseases after removal of the MHC region.

The percentage of SNPs with $p < \alpha$ (red circles) is plotted for their distance to the closest gene and values have been smoothed using a 50 kb sliding window (blue line). The light blue area represents the distribution expected by chance (95% confidence intervals) based on 100,000 permutations of the disease status. The purple triangle represents the proportion of SNPs with $p < \alpha$ in coding exons. The MHC region was excluded for all plots. The plots on the left and the right side show the same data at different scaling.
