# Supplementary material for: Exome localization of complex disease association signals
Source: BMC Genomics. 2011 Feb 1;12:92. doi: 10.1186/1471-2164-12-92 (PMC3045337; doi:10.1186/1471-2164-12-92)
Supplement: Additional File 2 — Enrichment Tables for all Diseases and Thresholds. This file presents the enrichment tables for all seven diseases and thresholds α. Enrichment tables after removal of the MHC region are provided for RA, T1D and the combined enrichment of all seven diseases. [file 1471-2164-12-92-S2.PDF]

Additional File 2: Supplementary Tables

Table S1: Number of SNPs per window in the observed and permuted datasets (Crohn's Disease)

CD		observed				95% confidence interval		
	Distance	total	p<0.1	p<0.01	p<0.001	p<0.1	p<0.01	p<0.001
Upstream	-100	2883	277	31	2	246-333	16-44	0-8
	-90	3092	286	30	11	266-355	18-46	0-8
	-80	3402	351	28	5	294-387	20-50	0-9
	-70	3966	396	51	7	347-447	25-56	0-10
	-60	4664	450	42	11	412-521	30-65	0-11
	-50	5324	569	49	6	475-591	36-73	1-12
	-40	6444	645	62	9	581-709	45-86	1-14
	-30	7651	823	92	22	696-834	55-100	2-15
	-20	10451	1099	131	28	963-1127	79-132	4-19
	-10	14095	1452	164	31	1311-1508	110-173	6-25
Gene	0	152344	15511	1649	284	14698-15747	1353-1687	109-201
Exon	0	2876	328	40	12	253-323	18-41	0-7
Downstream	10	15064	1550	208	38	1403-1609	119-184	6-26
	20	8954	893	110	29	818-973	66-115	3-18
	30	6860	668	75	18	620-752	48-91	1-14
	40	5555	588	70	11	496-617	37-76	1-12
	50	4612	444	36	5	408-516	30-64	0-11
	60	4088	413	38	4	358-461	26-58	0-10
	70	3507	339	32	1	304-399	21-51	0-9
	80	3262	330	35	8	281-373	19-48	0-9
	90	2882	300	30	7	246-332	16-44	0-8
	100	2601	264	46	10	220-301	14-40	0-8
total		274577	27976	3049	559	25443-28892	2183-3283	134-457

Additional File 2: Supplementary Tables:
B Lehne, CM Lewis, T Schlitt: Exome Localization of Complex Disease Association Signals; BMC Genomics

Table S2: Number of SNPs per window in the observed and permuted datasets (Type 1 Diabetes)

T1D		observed				95% confidence interval		
	**Distance**	**total**	**p<0.1**	**p<0.01**	**p<0.001**	**p<0.1**	**p<0.01**	**p<0.001**
**Upstream**	**-100**	2881	299	40	5	246-332	16-44	0-8
	**-90**	3083	310	27	1	265-353	18-46	0-8
	**-80**	3398	380	25	3	294-387	20-50	0-9
	**-70**	3962	398	47	10	347-447	25-57	0-10
	**-60**	4658	473	60	22	411-521	30-65	0-11
	**-50**	5327	531	96	17	475-592	35-73	1-12
	**-40**	6431	629	94	33	580-708	45-86	1-14
	**-30**	7643	773	116	58	696-835	55-100	2-16
	**-20**	10451	1146	220	103	963-1128	79-132	4-19
	**-10**	14085	1473	292	117	1310-1507	110-173	6-25
**Gene**	**0**	152114	15850	2086	472	14677-15729	1355-1684	109-200
**Exon**	**0**	2873	352	69	28	253-323	18-41	0-7
**Downstream**	**10**	15065	1641	300	138	1405-1609	119-184	6-26
	**20**	8940	1014	206	100	816-972	66-115	3-18
	**30**	6848	687	108	49	619-751	48-91	2-14
	**40**	5561	571	82	30	497-617	37-76	1-12
	**50**	4612	462	49	18	408-516	30-64	0-11
	**60**	4086	391	46	9	358-461	26-58	0-10
	**70**	3500	321	33	7	304-397	21-51	0-9
	**80**	3260	352	36	6	280-373	19-48	0-9
	**90**	2880	278	24	1	246-332	16-44	0-8
	**100**	2597	271	37	4	220-301	14-40	0-8
**total**		274255	28602	4093	1231	25417-28868	2184-3281	135-457

Table S3: Number of SNPs per window in the observed and permuted datasets (Type 1 Diabetes) after removal of the MHC region.

T1D		observed				95% confidence interval		
	Distance	total	p<0.1	p<0.01	p<0.001	p<0.1	p<0.01	p<0.001
Upstream	-100	2881	297	39	4	245-332	16-44	0-8
	-90	3083	310	27	1	265-353	18-46	0-8
	-80	3398	378	24	2	294-386	20-50	0-9
	-70	3962	391	43	6	346-446	25-56	0-10
	-60	4658	454	43	5	409-519	30-65	0-11
	-50	5327	514	82	6	472-589	35-73	1-12
	-40	6431	598	73	17	576-703	44-85	1-14
	-30	7643	713	69	16	688-827	54-99	2-15
	-20	10451	1034	118	18	950-1113	78-130	3-19
	-10	14085	1351	186	29	1294-1490	109-171	6-24
Gene	0	152114	15602	1891	300	14646-15699	1352-1681	109-199
Exon	0	2847	326	45	8	250-320	18-40	0-7
Downstream	10	15065	1512	197	47	1385-1587	117-182	6-26
	20	8940	914	123	26	806-959	65-114	3-17
	30	6848	631	70	14	613-744	48-90	1-14
	40	5561	544	57	9	494-614	37-75	1-12
	50	4612	446	34	6	406-514	30-64	0-11
	60	4086	381	37	2	357-459	25-58	0-10
	70	3500	316	28	5	303-396	21-51	0-9
	80	3260	350	34	4	280-373	19-48	0-9
	90	2880	277	23	0	245-332	16-44	0-8
	100	2597	269	36	3	219-301	14-40	0-8
total		271382	27282	3234	520	25293-28736	2173-3266	133-453

Table S4: Number of SNPs per window in the observed and permuted datasets (Rheumatoid Arthritis)

**RA**				**observed**		**95% confidence interval**		
	**Distance**	**total**	**p<0.1**	**p<0.01**	**p<0.001**	**p<0.1**	**p<0.01**	**p<0.001**
**Upstream**	**-100**	2881	286	31	1	246-332	16-44	0-8
	**-90**	3085	331	29	2	265-354	18-46	0-8
	**-80**	3404	320	34	2	295-388	20-50	0-9
	**-70**	3953	368	55	13	346-446	25-56	0-10
	**-60**	4655	495	53	5	411-521	30-65	0-11
	**-50**	5331	522	59	18	476-592	36-73	1-12
	**-40**	6432	735	92	19	579-708	45-86	1-14
	**-30**	7646	848	124	36	695-835	55-100	2-15
	**-20**	10454	1184	179	52	963-1128	79-132	4-19
	**-10**	14083	1529	205	58	1310-1508	110-173	6-25
**Gene**	**0**	152227	15674	1724	285	14686-15736	1354-1686	109-200
**Exon**	**0**	2874	319	43	14	253-323	18-41	0-7
**Downstream**	**10**	15059	1647	230	55	1404-1608	119-184	6-26
	**20**	8940	973	150	44	817-972	66-115	3-17
	**30**	6847	730	98	34	618-751	48-91	1-14
	**40**	5556	600	68	19	496-616	37-76	1-12
	**50**	4621	434	52	7	408-517	30-65	0-11
	**60**	4087	424	43	8	358-461	25-58	0-10
	**70**	3501	336	33	12	304-398	21-51	0-9
	**80**	3259	326	37	4	280-373	19-49	0-9
	**90**	2886	275	33	4	246-333	16-44	0-8
	**100**	2601	251	31	1	220-301	14-40	0-8
**total**		274382	28607	3403	693	25423-28878	2183-3284	134-455

Table S5: Number of SNPs per window in the observed and permuted datasets (Rheumatoid Arthritis) after removal of the MHC region.

RA		observed				95% confidence interval		
	**Distance**	**total**	**p<0.1**	**p<0.01**	**p<0.001**	**p<0.1**	**p<0.01**	**p<0.001**
**Upstream**	**-100**	2881	285	31	1	245-332	16-44	0-8
	**-90**	3085	331	29	2	265-354	18-46	0-8
	**-80**	3404	317	34	2	294-388	20-50	0-9
	**-70**	3953	358	48	6	345-444	25-56	0-10
	**-60**	4655	487	52	4	409-519	30-65	0-11
	**-50**	5331	503	43	9	473-589	35-73	1-12
	**-40**	6432	706	74	6	576-703	44-85	1-14
	**-30**	7646	802	93	9	688-827	54-99	2-15
	**-20**	10454	1097	128	15	950-1112	78-130	3-19
	**-10**	14083	1430	146	20	1295-1490	109-171	6-24
**Gene**	**0**	152227	15495	1600	187	14654-15705	1351-1683	109-200
**Exon**	**0**	2848	303	33	4	251-320	18-40	0-7
**Downstream**	**10**	15059	1554	178	22	1385-1587	117-182	6-26
	**20**	8940	909	105	10	806-960	65-114	3-17
	**30**	6847	695	74	15	612-744	48-90	1-14
	**40**	5556	580	59	15	494-613	37-75	1-12
	**50**	4621	421	47	6	406-515	30-64	0-11
	**60**	4087	418	40	7	357-460	25-58	0-10
	**70**	3501	328	25	4	303-397	21-51	0-9
	**80**	3259	326	37	4	280-373	19-49	0-9
	**90**	2886	275	33	4	246-333	16-44	0-8
	**100**	2601	251	31	1	220-301	14-40	0-8
**total**		271508	27568	2907	349	25303-28746	2172-3269	133-454

Table S6: Number of SNPs per window in the observed and permuted datasets (Hypertension)

HT			observed			95% confidence interval		
	Distance	total	p<0.1	p<0.01	p<0.001	p<0.1	p<0.01	p<0.001
Upstream	-100	2884	278	26	5	246-333	16-44	0-8
	-90	3094	305	19	3	266-355	18-46	0-8
	-80	3400	308	20	3	294-387	20-50	0-9
	-70	3957	371	47	6	347-446	24-57	0-10
	-60	4659	410	43	8	412-522	30-65	0-11
	-50	5341	563	41	2	476-593	36-73	1-12
	-40	6427	604	49	5	579-708	45-86	1-14
	-30	7645	811	80	13	695-835	55-100	2-15
	-20	10436	1074	111	19	962-1126	79-132	3-19
	-10	14075	1542	187	22	1309-1506	110-173	6-25
Gene	0	152229	15049	1565	182	14686-15736	1353-1687	109-200
Exon	0	2871	330	36	6	253-322	18-41	0-7
Downstream	10	15050	1526	148	23	1403-1607	119-184	6-26
	20	8947	861	84	8	818-972	66-115	3-18
	30	6836	624	69	7	617-750	48-90	1-14
	40	5556	594	68	5	496-616	37-76	1-12
	50	4620	456	38	4	409-517	30-65	0-11
	60	4089	387	29	2	358-461	26-58	0-10
	70	3503	324	20	2	304-398	21-51	0-9
	80	3257	380	45	8	280-373	19-49	0-9
	90	2879	291	24	2	246-332	16-44	0-8
	100	2597	268	23	3	220-301	14-40	0-7
total		274352	27356	2772	338	25423-28874	2182-3285	133-455

Additional File 2: Supplementary Tables:
B Lehne, CM Lewis, T Schlitt: Exome Localization of Complex Disease Association Signals; BMC Genomics

Table S7: Number of SNPs per window in the observed and permuted datasets (Coronary Artery Disease)

**CAD**		observed				95% confidence interval		
	**Distance**	**total**	**p<0.1**	**p<0.01**	**p<0.001**	**p<0.1**	**p<0.01**	**p<0.001**
**Upstream**	**-100**	2878	265	23	2	245-331	16-44	0-8
	**-90**	3087	353	48	11	265-354	18-46	0-8
	**-80**	3410	343	35	5	295-388	20-50	0-9
	**-70**	3966	412	41	4	347-447	25-57	0-10
	**-60**	4664	526	53	10	412-522	30-65	0-11
	**-50**	5331	582	57	8	475-593	36-73	1-12
	**-40**	6437	637	79	12	580-708	45-86	1-14
	**-30**	7645	786	92	17	695-834	55-100	2-15
	**-20**	10450	1067	114	16	963-1128	79-132	4-19
	**-10**	14064	1451	132	14	1308-1505	110-173	6-25
**Gene**	**0**	152106	15279	1449	149	14677-15727	1353-1686	109-200
**Exon**	**0**	2866	319	36	5	253-322	18-40	0-7
**Downstream**	**10**	15060	1477	124	18	1403-1608	119-184	6-26
	**20**	8948	896	87	7	818-973	66-115	3-18
	**30**	6844	715	75	10	619-751	48-90	2-14
	**40**	5555	543	49	7	496-616	37-76	1-12
	**50**	4615	470	40	1	408-516	30-64	0-11
	**60**	4087	360	31	4	358-461	25-58	0-10
	**70**	3493	307	29	4	303-397	21-51	0-9
	**80**	3256	281	23	2	280-373	19-48	0-9
	**90**	2885	287	34	6	246-333	16-44	0-8
	**100**	2606	292	27	0	221-302	14-40	0-8
**total**		274253	27648	2678	312	25414-28867	2182-3282	135-456

Table S8: Number of SNPs per window in the observed and permuted datasets (Type 2 Diabetes)

T2D		observed				95% confidence interval		
	Distance	total	p<0.1	p<0.01	p<0.001	p<0.1	p<0.01	p<0.001
Upstream	-100	2876	254	25	8	246-332	16-44	0-8
	-90	3081	280	15	1	265-353	18-46	0-8
	-80	3397	357	22	3	294-386	20-50	0-9
	-70	3952	409	42	4	347-446	25-57	0-10
	-60	4640	455	44	3	410-519	30-65	0-11
	-50	5320	511	58	6	474-590	36-73	1-12
	-40	6419	642	49	3	578-706	45-86	1-14
	-30	7621	813	88	10	693-832	55-99	2-15
	-20	10421	1115	123	15	960-1125	79-131	4-19
	-10	14064	1434	150	8	1308-1505	110-173	6-25
Gene	0	151876	15466	1634	208	14656-15701	1351-1683	108-200
Exon	0	2863	292	33	1	252-322	18-40	0-7
Downstream	10	15022	1625	166	13	1400-1604	118-184	6-26
	20	8915	911	88	6	815-969	66-115	3-17
	30	6838	675	60	5	618-750	48-91	1-14
	40	5547	560	57	8	496-615	37-76	1-12
	50	4614	458	38	2	408-516	30-65	0-11
	60	4082	448	47	5	358-461	25-58	0-10
	70	3488	354	45	9	303-396	21-51	0-9
	80	3255	324	28	2	280-373	19-48	0-9
	90	2876	306	25	0	245-332	16-44	0-8
	100	2594	268	44	5	220-301	14-40	0-7
total		273761	27957	2881	325	25374-28812	2179-3279	133-454

Additional File 2: Supplementary Tables:
B Lehne, CM Lewis, T Schlitt: Exome Localization of Complex Disease Association Signals; BMC Genomics

Table S9: Number of SNPs per window in the observed and permuted datasets (Bipolar Disorder)

BD				observed		95% confidence interval		
	**Distance**	**total**	**p<0.1**	**p<0.01**	**p<0.001**	**p<0.1**	**p<0.01**	**p<0.001**
**Upstream**	**-100**	2872	285	25	2	245-331	16-44	0-8
	**-90**	3071	331	36	1	264-352	18-46	0-8
	**-80**	3386	338	30	3	293-386	20-50	0-9
	**-70**	3949	402	39	10	346-446	25-56	0-10
	**-60**	4638	444	56	10	409-519	30-65	0-11
	**-50**	5305	539	70	4	473-589	35-73	1-12
	**-40**	6414	637	75	7	578-705	44-86	1-14
	**-30**	7617	775	95	6	692-831	55-99	2-15
	**-20**	10407	1116	126	18	959-1122	79-131	4-19
	**-10**	14036	1471	177	23	1305-1501	110-172	6-24
**Gene**	**0**	151714	15440	1718	179	14634-15679	1348-1680	108-199
**Exon**	**0**	2861	306	49	2	252-321	18-40	0-7
**Downstream**	**10**	15000	1605	158	13	1398-1602	118-183	6-26
	**20**	8911	909	81	13	814-969	65-115	3-17
	**30**	6834	678	55	9	617-750	48-91	2-14
	**40**	5536	524	45	5	494-614	37-76	1-12
	**50**	4600	416	38	9	407-515	30-64	0-11
	**60**	4067	383	26	2	356-459	25-58	0-10
	**70**	3488	315	27	2	302-396	21-51	0-9
	**80**	3254	324	24	1	280-372	19-48	0-9
	**90**	2871	268	23	1	245-331	16-44	0-8
	**100**	2587	253	22	0	219-300	14-40	0-7
**total**		273418	27759	2995	320	25330-28769	2173-3272	134-452

Table S10: Number of SNPs per window in the observed and permuted datasets (combined diseases). Numbers were averaged over all seven diseases.

mean		observed				95% confidence interval		
	**Distance**	**total**	**p<0.1**	**p<0.01**	**p<0.001**	**p<0.1**	**p<0.01**	**p<0.001**
**Upstream**	**-100**	2,879	278	29	4	246-332	16-44	0-8
	**-90**	3,085	314	29	4	265-354	18-46	0-8
	**-80**	3,400	342	28	3	294-387	20-50	0-9
	**-70**	3,958	394	46	8	347-446	25-57	0-10
	**-60**	4,654	465	50	10	411-521	30-65	0-11
	**-50**	5,326	545	61	9	475-591	36-73	1-12
	**-40**	6,429	647	71	13	579-707	45-86	1-14
	**-30**	7,638	804	98	23	695-834	55-100	2-15
	**-20**	10,439	1,114	143	36	962-1126	79-132	4-19
	**-10**	14,072	1,479	187	39	1,309-1,506	110-173	6-25
**Gene**	**0**	152,087	15,467	1,689	251	14,673-15,722	1,352-1,685	109-200
**Exon**	**0**	2,869	321	44	10	253-322	18-41	0-7
**Downstream**	**10**	15,046	1,582	191	43	1,402-1,607	119-184	6-26
	**20**	8,936	922	115	30	817-971	66-115	3-18
	**30**	6,844	682	77	19	618-751	48-91	1-14
	**40**	5,552	569	63	12	496-616	37-76	1-12
	**50**	4,613	449	42	7	408-516	30-64	0-11
	**60**	4,084	401	37	5	358-461	25-58	0-10
	**70**	3,497	328	31	5	303-397	21-51	0-9
	**80**	3,258	331	33	4	280-373	19-48	0-9
	**90**	2,880	286	28	3	246-332	16-44	0-8
	**100**	2,598	267	33	3	220-301	14-40	0-8
**total**		383,499	38,721	4,166	648	25,404-28,851	2,181-3,282	134-456

Table S11: Number of SNPs per window in the observed and permuted datasets (combined diseases) after removal of the MHC region. Numbers were averaged over all seven diseases.

mean			observed			95% confidence interval		
	**Distance**	**total**	**p<0.1**	**p<0.01**	**p<0.001**	**p<0.1**	**p<0.01**	**p<0.001**
**Upstream**	**-100**	2,879	277	29	3	245-332	16-44	0-8
	**-90**	3,085	314	29	4	265-354	18-46	0-8
	**-80**	3,400	342	28	3	294-387	20-50	0-9
	**-70**	3,958	391	44	6	346-445	25-56	0-10
	**-60**	4,654	460	47	7	409-519	30-65	0-11
	**-50**	5,326	537	57	6	472-588	35-73	1-12
	**-40**	6,429	633	65	8	575-703	44-85	1-14
	**-30**	7,638	779	86	13	687-826	54-99	2-15
	**-20**	10,439	1,072	119	17	949-1111	78-130	3-19
	**-10**	14,072	1,427	161	20	1293-1488	109-171	6-24
**Gene**	**0**	152,087	15,376	1,639	212	14642-15692	1350-1682	108-200
**Exon**	**0**	2,843	314	39	5	250-319	18-40	0-7
**Downstream**	**10**	15,046	1,530	166	24	1383-1585	117-182	6-26
	**20**	8,936	887	95	13	806-959	65-114	3-17
	**30**	6,844	660	67	11	612-744	48-90	1-14
	**40**	5,552	558	57	8	493-613	37-75	1-12
	**50**	4,613	443	39	5	406-514	30-64	0-11
	**60**	4,084	397	35	4	357-459	25-58	0-10
	**70**	3,497	326	29	4	303-396	21-51	0-9
	**80**	3,258	331	32	4	280-373	19-48	0-9
	**90**	2,880	286	27	3	245-332	16-44	0-8
	**100**	2,598	266	33	3	219-301	14-40	0-8
**total**		271,273	27,293	2,885	381	25283-28720	2170-3266	132-453
